# Influence of Life Style Factors on Barrett's Oesophagus

**DOI:** 10.1155/2014/408470

**Published:** 2014-05-26

**Authors:** A. Horna Strand, T. Franzén

**Affiliations:** ^1^Department of Transplantation Surgery, Department of Clinical Science, Intervention and Technology (CLINTEC), Karolinska Institutet, Gastrocentrum, Karolinska University Hospital, 14186 Stockholm, Sweden; ^2^Department of Surgery, the Faculty of Health Sciences, Linköping University, 58183 Linköping, Sweden

## Abstract

*Background*. Since the incidence of adenocarcinoma of the oesophagus is rising, the prognosis is poor, and surveillance programs are expensive and mostly cost ineffective, there is a need to increase the knowledge of risk factors in Barrett's oesophagus and oesophageal cancer in order to be able to give attention to medical prevention and/or surveillance programs. *Aim*. To study if there is a correlation between the development of Barrett's oesophagus and GOR (gastro oesophageal reflux), family history of GOR, and life style factors, such as alcohol, smoking habits, and mental stress. *Methods*. Fifty-five consecutively selected patients with Barrett's oesophagus (BO) examined at Linköping University Hospital's Oesophageal Laboratory were matched by sex, age, and duration of reflux symptoms with 55 GOR patients without Barrett's oesophagus at the Oesophageal Laboratory. The medical charts in respective groups were examined for comparison of life style factors, mental stress, medication, duration of gastroesophageal acid reflux at 24 hr-pH-metry, and incidence of antireflux surgery and of adenocarcinoma of the oesophagus (ACO). Also, potential gender differences and diagnosis of ACO were studied. *Results*. Mean percentage reflux time on 24 hr-pH-metry was higher for the Barrett's oesophagus group, 18% for women and 17% for men compared to 4% for women and 4% for men in the control group (*P* < 0.05). Family history of GOR was more frequent in Barrett's oesophagus patients (62%) than in the control group (35%) (*P* < 0.05). Male patients with Barrett's oesophagus had medical therapy for their GOR symptoms to a higher extent (38%) than male controls (65%) (*P* < 0.05). No difference was found in the number of tobacco users or former tobacco users between Barrett's oesophagus patients and controls. Barrett's oesophagus patients had the same level of alcohol consumption and the same average BMI as the control subjects. Female patients with Barrett's oesophagus rated themselves as more mentally stressed (67%) than the female controls (38%) (*P* < 0.05). In the five-year medical chart follow-up, five of 55 patients developed adenocarcinoma among the Barrett's oesophagus patients, none in the control group. *Conclusions*. Long reflux time and family clustering of GOR seem to influence the development of Barrett's oesophagus. Smoking habits, alcohol consumption and BMI do not seem to have any impact on the development of Barrett's oesophagus.

## 1. Introduction

The understanding of Barrett's oesophagus (BO) has improved in recent years, but still much remains unknown [1, 2]. BO would be of little importance if not for its well-recognized association with adenocarcinoma of the oesophagus (ACO) [1, 3]. BO is the only known precursor of ACO and therefore has major public health care economic implications [1, 4]. ACO's incidence is low but increases more rapidly than any other type of cancer [5, 6]. Its prognosis is poor [7].

There is a lack of robust data on risk factors involved in the BO transformation [4, 8].

Several risk factors have been studied but the studies show partly contradictory results. Most published BO studies are small (Jochem 6 patients, Neumann 16 patients, and Gillen 24 patients) [9–11] or have no control group (Katz 102 patients) [12]. Much of the BO knowledge is known through ACO studies. However, no study found on PubMed has a matched control group concerning gender, age, and duration of GOR symptoms.

Shaheen and Ransohoff [5] and Lagergren et al. [13] have found the risk of developing BO to increase with frequency and duration of GOR symptoms but not Neumann and Cooper [10] and Gillen et al. [11]. Lagergren et al. and Vaughan et al. have shown a higher incidence of BO in GOR patients who use alcohol or tobacco and have a higher BMI (body mass index) than GOR patients without BO [13, 14]. Concerning BMI, Kabat and Zhang found contradicting results [13, 14]. Jochem et al. and Romero et al. have found family-related clustering of BO [9, 15]. One report has suggested that fundoplication might be more effective than medical therapy for preventing neoplastic complications of GOR [12]. Two studies found no correlation between ACO and antisecretory medical therapy [16, 17]. As stated earlier, none of the studies referred to include a large number of patients studied over a short study span at a single center with a control group matched by age, gender, and duration of GOR symptoms.

Incidence rates for BO are highest among middle-aged males and increase with age. Two-thirds of BO patients are men [13]. It has been estimated that the mean age for BO diagnosis is 63 years [18]. BO has been found in approximately 6–15 percent of patients undergoing endoscopy for symptoms of GOR and in one percent or less of unselected patient populations undergoing endoscopy [15, 18]. In most individuals with BO, the condition remains undiagnosed [19]. One study showed that four out of 64 (6%) patients with ACO had received their BO diagnosis before the discovering of ACO [20]. Another study showed that BO patients developed ACO at an annual rate of 0.4 percent, compared to GOR patients whose rate was calculated to 0.07 percent [21]. The identification of high-risk groups for progression to ACO might make screening and surveillance a cost- effective practice [4, 5]. The aim of this study is to study if life style factors, such as alcohol, cigarette smoking, self-evaluated mental stress, family history of GOR symptoms, or duration of acid reflux, will influence the development of Barrett's oesophagus.

## 2. Material and Methods

The 55 first patients with Barrett's oesophagus (BO) (34 males, 21 females), who were examined at the Oesophageal Laboratory in the Surgery Department of Linköping University Hospital during a nine-month period, were matched by sex, age (±3 years), and duration of reflux symptoms with GOR patients that attended the Oesophageal Laboratory during the same time period. The study and control group were found by going through the questionnaires from telephone interviews that were part of the standard follow-up after examination at the Linköping University Hospital Oesophageal Laboratory. The questionnaires were archived in the patients' medical charts. The medical charts were reviewed between August and December 2010. The questionnaire consisted of questions about medical treatment, GOR symptoms, quality of life, self-evaluated mental stress, BMI, family history of GOR, coffee, tobacco, and alcohol consumption. The study group and control group were not contacted; information was only collected from their medical charts. The Linköping University Hospital Oesophageal Laboratory has patients from the county of Östergötland (400 000 inhabitants) and mostly from the city of Linköping (140 000 inhabitants).

The inclusion criteria were documented GOR (defined as heart burn, regurgitation, swallowing difficulties, acid reflux, and so forth, that by a doctor was described as GOR symptoms in the patient's medical chart) and the diagnosis BO for the study group. The BO definition used in this study is the most commonly used, that is, columnar epithelium extending ≥3 cm above the gastroesophageal junction diagnosed at endoscopy [5]. In all patients with Barrett's oesophagus, the diagnosis was confirmed by histology. None of the patients with Barrett's oesophagus had strictures, dysplasia, or adenocarcinoma at the time of diagnosis. The Oesophageal Laboratory at the Surgery Department at Linköping University Hospital managed all the 24 hr. ambulatory pH recordings. Proton pump inhibitors were suspended two weeks before being investigated at the Oesophageal Laboratory.

The control group's GOR symptoms were documented in the same manner as for the study group. The duration of GOR symptoms from the questionnaire was controlled to be the same in the medical chart (patients with GOR symptoms often become insensitive to reflux when they develop Barrett's oesophagus). The severity of the reflux was not measured. Patients, in whom mental disorders, drug abuse, and/or dementia were described in their journals or in the telephone questionnaires, were excluded. No BO patient was excluded but 4 potential control persons were excluded for these reasons. Linköping has approximately the same socioeconomic distribution as the average Swedish city.

The study group consisted of 21 women and 34 men. The mean age of the women and men in the study and control group was 70.4, SD 14.1, respectively, 64.1, SD 11.0. The groups' medical charts were examined for comparison of life style factors, mental stress, medication, duration of gastroesophageal acid reflux at 24 hr. pH-metry, the average age of BO diagnosis, potential gender differences, and the incidence of fundoplication operation and ACO was studied in the charts. Also, the duration from BO diagnosis to fundoplication operation and to the development of ACO was examined. Paired *t*-test was used for statistical analysis. The parameters are expressed as mean ± SD (standard deviation). The significance level was set at *P* < 0.05. Informed consent to use the information unanimous on a group level was obtained from each person when undertaking the follow-up telephone interviews. The Ethics Committee of the Linköping University Hospital has approved the study.

## 3. Results

The mean percentage reflux time of BO women and BO men was significantly higher than that of the male and female controls, 18 and 17 percent compared to 4 and 4 percent (*P* < 0.05). The male study group had done a higher number of 24 hr. pH-metry than the male control group (*P* < 0.05). Family history of GOR symptoms was found in 53 of 110 patients. It existed more in the study group for the men's families than in the male control group (*P* < 0.05). Paternal hereditary history was found in a lower degree in the male study group than in the male control group (*P* < 0.05). The male study group used more medical treatment for GOR symptoms than the male control group (*P* < 0.05). Sixty-one out of 110 were or had been tobacco users. There was no significant difference between the BO patients and the control subjects concerning the use of tobacco. No correlation was found between BO and alcohol consumption. There was no significant difference concerning BMI or coffee consumption.

The female study group rated themselves as more mentally stressed than the female control group (*P* < 0.05). For the complete results, see Tables 1 and 2.

BO patients developed GOR symptoms at the age of 23 (*P* < 0.05). The time before seeking medical attention for GOR symptoms was 10 years, which was significantly lower for BO women and BO men (*P* < 0.05). The mean age of having a fundoplication operation for BO patients was 52 years, that is, 29 years after the BO patients developed GOR symptoms and 19 years after seeking medical attention. The study group had made a fundoplication operation to a higher extent than the control group (*P* < 0.05). Also, the male study group had the operation at an earlier age than the control group for men (*P* < 0.05). The average age when receiving the BO diagnosis was 53 years. The BO patients had GOR symptoms for approximately 30 years before developing BO. They sought medical attention 20 years after receiving the BO diagnosis. One year before BO diagnosis, the patients had a fundoplication operation. Thirty-six years after developing GOR symptoms five out of 55 patients (2 women, 3 men) developed adenocarcinoma of the oesophagus. In the control group no one developed ACO. The patients were given the ACO diagnosis 27 years after seeking medical care for GOR symptoms for the first time. The average age for developing cancer was 65 years for the two women and 54 years for the three men. The women got the BO diagnosis 11 years before receiving the cancer diagnosis while the men got the diagnosis only two years before the cancer diagnosis.

## 4. Discussion

This study showed no correlation between BO and alcohol, tobacco smoking, and high BMI. Concerning BMI, this study's result supports the findings of Kabat and Zhang but not Lagergren and Vaughan's results [13, 14, 22, 23]. Kabat's study group was large, N 173, but consisted only of men that were matched by gender and age ±5 years. Also, all had ACO. Zhang also studied ACO patients and not BO patients. In the Zhang study the patients were matched by age and sex. Lagergren's study consisted of 189 nonmatched patients. Vaughan studied 298 ACO patients matched for age ±5 years and gender.

The incidence of family clustering was significantly higher among BO patients. This was also found in other studies [9, 15], but their study populations were smaller than ours, 6 and 40 compared to 55 BO patients.

In our study BO women rated themselves as experiencing more mental stress than the BO men or the control group.

The mean age of developing BO is of value for deciding when to do control gastroscopies and for designing the patient's follow-up plan. With the exception of mean age of BO diagnosis, we have not found that these data have been studied in a published study before.

Our study found that BO patients sought hospital care earlier than GOR patients without BO. The reason for this might be that the symptoms of BO patients are greater than those of GOR patients without Barrett's oesophagus. The much higher acid level of BO patients might support such a hypothesis. Also, this might explain why BO men had done more 24 hr. pH-metries and were operated to a higher extent and at an earlier age. However, although the BO men were operated earlier and more frequently there was no significant difference between BO women and the men's mean percentage reflux time. Possibly this variation indicates that the symptoms of GOR are not only correlated with acid reflux or that there is a different tolerance for acid between men and women.

As mentioned above, this study found that the BO patients had much higher mean percentage reflux time than GOR patients without Barrett's oesophagus. These results match those of Shaheen and Ransohoff [5] and Lagergren et al. [13] but not Neumann and Cooper [10] and Gillen et al. [11]. Shaheen's study is a case study including three cases. As noted above, Lagergren's study consisted of 189 nonmatched ACO patients. Gillen's study group consisted of 24 BO patients matched with 24 esophagitis patients. However, the BO patients were not matched for age, gender, and duration of GOR symptoms. Neumann's study consisted of 16 BO patients comparable in age and gender.

Fundoplication might be more effective than medical therapy for preventing both peptic and neoplastic complications of GOR [12]. However, in this study, BO patients were operated to a significantly higher extent and the BO men at a significantly younger age. Men with BO used medical therapy more frequently than female BO patients and patients with GOR symptoms but no BO. However, no correlation between ACO and antisecretory agents has been found [16, 17]. Chow's study consisted of 196 ACO patients matched by gender and age ±5 years. Farrow's study consisted of 293 ACO patients matched by gender and age ±5 years. Katz study consisted of 102 BO patients with no control group with data collected over a 24-year span. In the beginning of the Katz' study period the BO diagnosis was not commonly used [12].

In our study the GOR symptoms started at the age of 24 and the average BO diagnosis was set at 53 years which was 14 years and 7 years earlier than in a study previous [18]. Cameron's study was large, *N* = 377, compared to 55 in this study, but had no control group. Since our study population and our ACO incidence were not large enough to study the incidence of ACO, we do not draw any conclusions of the 5 of 55 BO patients developing ACO. The strength of this study is that the study group is matched for gender, age, and duration of GOR symptoms. Therefore we believe that this study's results lay closer to the truth than results by studies with no control groups. Also, we believe that to study the development of ACO one should study Barrett Oesophagus patients in first hand, since the time for developing ACO is so long and that makes a retrospective ACO study less trustworthy.

An improvement of this study would be an analysis of the type and length of BO and the degree of GOR symptoms. The reason the severity of the patients' GOR symptoms was not noted was that this piece of information was not available in the patient's medical cart including not in the telephone interview questionnaire. Perhaps an approximation of the severity of GOR symptoms was measured through the 24 hr. pH-metry mean reflux time and by how fast the patients sought medical attention and were operated. However, many published studies have neither examined this nor matched their study population as discussed above. The study group could also be larger but BO patients are hard to study since many are undiagnosed and in accordance with the power analysis a study group of 55 patients was sufficient. Also, if the BO population was to be divided into subgroups based on grade of BO the groups would have been too small to draw conclusions from. The significant results of this study need to be examined in a larger study population but should be considered to be closer to the truth than the results of small studies with no matched control groups. The nonsignificant results are also important in designing better potential surveillance programs.

## Figures and Tables

**Table 1:** Results of study and control group.

	Study group			Control group		
	Women	Men	All	Women	Men	All
	(*N* = 21)	(*N* = 34)	(*N* = 55)	(*N* = 21)	(*N* = 34)	(*N* = 55)
Age	70	64	60	70	64	70
Age at GOR symptoms	25	22	23	24	21	23
Years before medical attention	10.2 ± 10*	9.1 ± 9*	9.5 ± 9	16.2 ± 11	15.1 ± 11	15.5 ± 11
Done a 24-H pH-metry (%)	95	97	96	100	91	94
Reflux time (%)	18.1 ± 19*	17.3 ± 13*	17.6 ± 16	3.8 ± 2	3.9 ± 3	3.8 ± 3
Operation (op) (%) (*N* = 55/110)	86*	74*	78	24	21	22
Age op (years)	55.1 ± 12	50.1 ± 11*	52.2 ± 11	57.8 ± 21	52.6 ± 12	54.8 ± 16
Op after years of symptoms (%)	30.4 ± 9	28.2 ± 10	28.8 ± 9	33.4 ± 12	31.2 ± 12	32.2 ± 11
Op after years of medical attention (%)	20.2 ± 5	19.1 ± 4	19.3 ± 4	17.2 ± 3	16.1 ± 2	16.7 ± 2
Reop (*N*)	1	6	7	2	0	2
Years between op	1	5	5	8	—	6
BMI	26.6 ± 4	26.4 ± 3	26.5 ± 3	26.4 ± 4	25.7 ± 2	26.0 ± 3
Tobacco (%) (*N* = 71)	57	62	60	57	76	69
Tobacco now (%) (*N* = 18)	33	33	33	17	19	18
Cigarettes (%) (*N* = 61)	100	90	94	100	73	82
Snuff (*N*) (*N* = 4)	0	1	1	0	3	3
Pipe (*N*) (*N* = 6)	0	1	1	0	5	5
Family history (%) (*N* = 53)	57*	65*	62	43	29	35
Maternal history (*N*) (*N* = 15)	4	6	10	4	1	5
Paternal history (*N*) (*N* = 15)	1	5*	6	2	7	9
Mental stress (%)	67*	76	72	8	88	69
Medical therapy (%) (*N* = 57)	52	38*	44	52	65	60
PPI (%) (*N* = 48)	91	92	92	73	82	79
Alcohol (%) (*N* = 89)	71	88	82	67	88	80
Coffee (cups/day)	3	3	3	2	3	2

*(*P* < 0.05), all other results non significant.

**Table 2:** Results of study group.

	Barrett		
	Women	Men	All
	(*N* = 21)	(*N* = 34)	(*N* = 55)
Diagnosis (age)	54.5 ± 15	52.1 ± 10	53.0 ± 12
Diagnosis after years of symptoms	29.8 ± 11	30.2 ± 9	29.6 ± 10
Diagnosis after years of medical attention	19.6 ± 4	21.1 ± 4	20.1 ± 4
Operation after years of diagnosis	0.6 ± 4	−2.0 ± 4	−0.8 ± 4
Age at developing cancer	65	54	60
Symptoms before cancer (years) (*N* = 5)	40.3 ± 5 (*N* = 2)	32.2 ± 20 (*N* = 3)	36.2 ± 14 (*N* = 5)
Seeking medical attention before cancer (years)	30.1 ± 14	23.1 ± 2	26.7 ± 8
Diagnosis before cancer (years)	10.5 ± 14	2.0 ± 2	5.4 ± 8
Fundoplication before cancer (*N*) (*N* = 3)	1	2	3

## Abbreviations

BO: Barrett's oesophagus
GOR: gastroesophageal reflux
ACO: adenocarcinoma of the oesophagus
BMI: body mass index.
